# A Homolog of Structural Maintenance of Chromosome 1 Is a Persistent Centromeric Protein Which Associates With Nuclear Pore Components in *Toxoplasma gondii*

**DOI:** 10.3389/fcimb.2020.00295

**Published:** 2020-07-02

**Authors:** Maria E. Francia, Sheila Bhavsar, Li-Min Ting, Matthew M. Croken, Kami Kim, Jean-Francois Dubremetz, Boris Striepen

**Affiliations:** ^1^Department of Cellular Biology, University of Georgia, Athens, GA, United States; ^2^Morsani College of Medicine, University of South Florida Health, Tampa, FL, United States; ^3^Pathology, Molecular and Cell Based Medicine, Mount Sinai Medical Center, New York, NY, United States; ^4^UMR 5235 CNRS, Université de Montpellier 2, Montpellier, France; ^5^Center for Tropical and Emerging Global Diseases, University of Georgia, Athens, GA, United States

**Keywords:** centromere, cohesin, nuclear pore, centrosome, cell division, microtubues, toxoplasma, toxoplasmosis

## Abstract

Apicomplexa are obligate intracellular parasites which cause various animal and human diseases including malaria, toxoplasmosis, and cryptosporidiosis. They proliferate by a unique mechanism that combines physically separated semi-closed mitosis of the nucleus and assembly of daughter cells by internal budding. Mitosis occurs in the presence of a nuclear envelope and with little appreciable chromatin condensation. A long standing question in the field has been how parasites keep track of their uncondensed chromatin chromosomes throughout their development, and hence secure proper chromosome segregation during division. Past work demonstrated that the centromeres, the region of kinetochore assembly at chromosomes, of *Toxoplasma gondii* remain clustered at a defined region of the nuclear periphery proximal to the main microtubule organizing center of the cell, the centrosome. We have proposed that this mechanism is likely involved in the process. Here we set out to identify underlying molecular players involved in centromere clustering. Through pharmacological treatment and structural analysis we show that centromere clustering is not mediated by persistent microtubules of the mitotic spindle. We identify the chromatin binding factor a homolog of structural maintenance of chromosomes 1 (SMC1). Additionally, we show that both TgSMC1, and a centromeric histone, interact with TgExportin1, a predicted soluble component of the nuclear pore complex. Our results suggest that the nuclear envelope, and in particular the nuclear pore complex may play a role in positioning centromeres in *T. gondii*.

## Introduction

**Figure 1:**
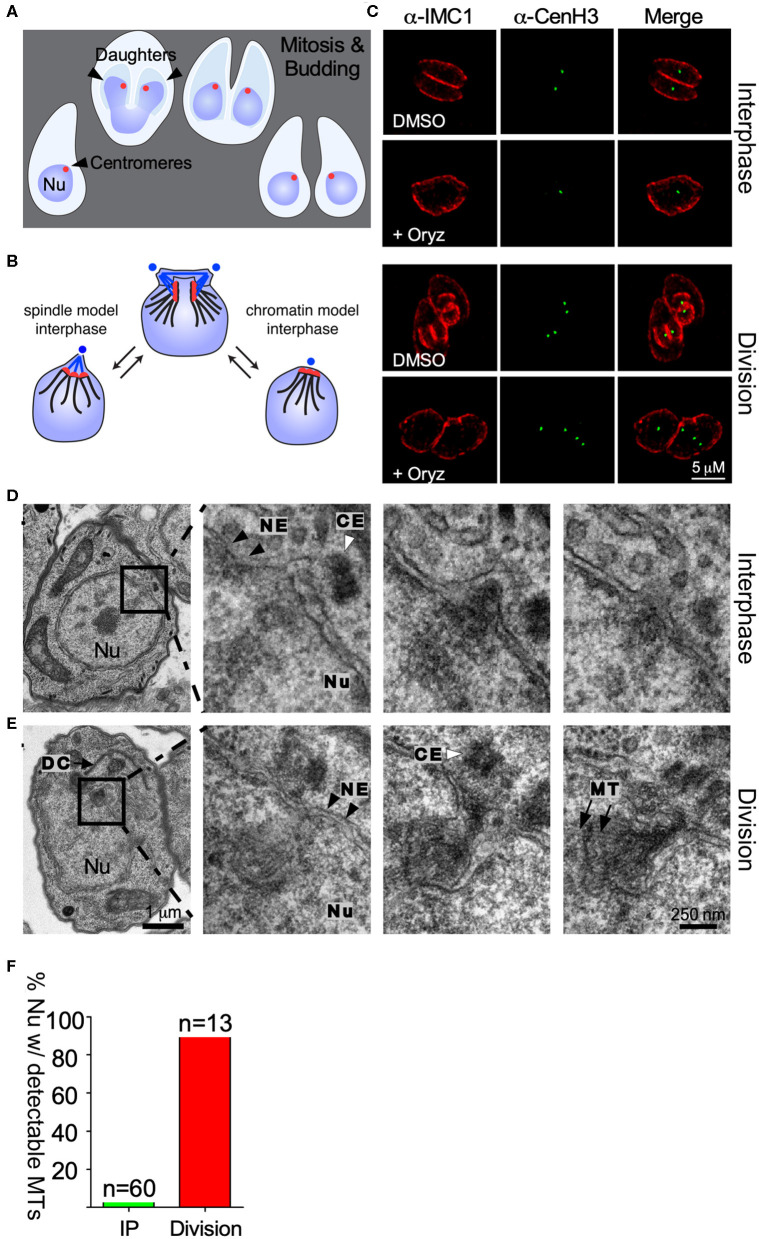
Centromere clustering is not mediated by spindle microtubules. **(A)** Apicomplexan parasite division schematic. Apicomplexa divide by closed mitosis and internal daughter cell assembly. Centromeres (represented as a red dot) remain clustered at the periphery of the nucleus throughout the cell cycle. **(B)** Alternative models proposed to explain centromere clustering. Blue dots represent the centrosome. Blue lines represent the mitotic spindle microtubules. Red dots represent the chromosomes' centromeres. **(C)** Parasites were treated with DMSO (control) or 2.5 mM Oryzalin, fixed and stained with anti-IMC1 and anti-CenH3. Both in DMSO and Oryzalin treated samples, interphase parasites display a single TgCenH3 dot corresponding to clustered centromeres. Both Oryzalin treated and untreated dividing parasites display duplicated TgCenH3 foci. In Oryzalin treated parasites daughter cell assembly and proper chromosome segregation is impaired as evidenced by the presence of multiple (>2) TgCenH3 foci within a single parasite. **(D)** TEM series through a parasite in interphase containing a single centrosome (CE, white arrowhead). Zoomed-in panels show consecutive series. Microtubules (MT) are not seen proximal to the centrosome (CE, white arrowheads) or the nuclear envelope (NE, black arrowheads) at the site of centromere sequestration. **(E)** TEM series through a dividing nucleus. A forming daughter cell (DC) is detectable proximal to the nucleus (Nu). The mitotic spindle organizes within the nuclear envelope (NE, black arrowheads). Zoomed-in panels show consecutive series. Microtubules (MT, black arrows) of the mitotic spindle are clearly visible in the proximity of the centrosome (CE, white arrowhead). Note that all serial sections were obtained from the same block, and thus were subject to identical fixation and post-fixation treatments. The scale for **(D,E)** is the same. **(F)** Parasites present in TEM serial sections were classified as “interphase” (IP) or “dividing”, depending on the presence of a single or a duplicated centrosome respectively, and scored for the presence of visible spindle microtubules.

Apicomplexa are obligate intracellular parasites that cause various animal and human diseases including malaria, toxoplasmosis, and cryptosporidiosis. Apicomplexan parasites invade and replicate within the cells of their hosts. Following intracellular replication, parasites lyse their host cell and invade a neighboring healthy cell thus perpetuating the infection. Apicomplexan parasites replicate by modes of division that differ from those used by their hosts (Francia and Striepen, 2014). Mammalian cells divide their nucleus by open mitosis in which the nuclear envelope breaks down, giving way to the mitotic spindle, and is immediately followed by cytokinesis (with few exceptions). Apicomplexa, however, combine semi-closed mitosis, in which the nuclear envelope remains practically intact, with the generation of multiple daughter cells by budding (Gubbels et al., 2008; Francia and Striepen, 2014; White and Suvorova, 2018) (schematically represented in Figure 1A). In apicomplexan cell division, daughter cells do not derive from fission but instead are formed *de novo* in the mother cell cytosol. The fundamental differences between these modes suggest that cell division could be a rich source of druggable targets to treat apicomplexan-caused diseases. However, many structural and regulatory aspects of apicomplexan cell division are not well-understood (White and Suvorova, 2018).

Direct visualization of chromosomes is impaired by the apparent lack of chromatin condensation throughout the cell cycle in the parasites' nuclei. Centromeres are typically a single location on a chromosome where kinetochore components, the point of attachment for microtubules of the mitotic spindle, assemble during mitosis. Centromeres are marked by the presence of a variant histone H3, known as CenH3 or CenPA. In the past, a *T. gondii* strain bearing an epitope tag in its CenH3 homolog, allowed visualization of the centromere-associated nucleosomes, allowing the mapping of the chromosomal position of the centromeres in *T. gondii*, and visualization of chromosomal dynamics during mitosis (Brooks et al., 2011). All stages observed in canonical eukaryotic mitosis (i.e., metaphase, anaphase, and telophase) are present in the *T. gondii* mitosis. However, all centromeres of *T. gondii* cluster into a single location at the periphery of the nucleus, not only during division but also outside of mitosis (Brooks et al., 2011). Moreover, the site of centromere clustering is intimately related to the position of the centrosome, the main microtubule organizing center (MTOC) of the cell, which nucleates microtubules of the mitotic spindle during division. Centromeres of *Plasmodium falciparum* were also shown to cluster in the proximity of its centrosome equivalent outside of mitosis (Hoeijmakers et al., 2012). Interestingly, while *T. gondii*, divides by endodyogeny, assembling two daughter cells every round of division*, P. falciparum*'s schizogony can yield hundreds of daughters per division. Thus, centromere clustering appears to be a widespread phenomenon among apicomplexans using different modes of division (Bunnik et al., 2019).

The molecular mechanisms mediating chromatin sequestration to defined nuclear territories and specialized sub-compartments are unknown in Apicomplexa. Here, we set out to identify novel molecular players involved in centromere positioning in *Toxoplasma gondii*.

## Methods

### Chromatin Immunoprecipitation

ChIP was performed as described in Wells and Farnham (2002); Gissot et al. (2007), and Brooks et al. (2011). Briefly, chromatin from SMC1-HA transgenic tachyzoites was cross-linked for 10 min with 1% formaldehyde at room temperature and purified after sonication yielding fragments of 500–1,000 bp. Chromatin was immunoprecipitated at 4°C overnight using a HA polyclonal antibody (Abcam ab9110) and washed extensively. The DNA was treated with proteinase K for 2 h and subsequently purified using the Qiagen PCR purification kit. Hundred nanogram of precipitated DNA was amplified using the DNA Genomeplex whole genome amplification kit (Sigma) and subsequently labeled using random primers coupled to a fluorochrome. Probes were hybridized to a tiled oligonucleotide array representing the complete *T. gondii* genome according to NimbleGen Systems procedures. The array was fabricated by NimbleGen Systems and contained 740,000 oligonucleotides representing version 4 of the ME49 genome with an approximate spacing of 80 bp between each oligonucleotide.

### Co-immunoprecipitation (Co-IP) and Mass Spectrometry Analysis

Approximately 1 × 10^9^ SMC1_YFP, RHΔKu80 or the HA-tagged lines generated in this study (TgImportin1-HA, TgExportin1-HA, TgSUN-HA), were collected by centrifugation, and washed once with PBS. Parasites were lysed by resuspension in hypotonic buffer (20 mM Hepes, 10 mM KCl, 400 mM Mannitol, 2 nM EDTA) supplemented with EDTA free protease inhibitor (Roche) to ~ 5 × 10^8^ parasites/ml, followed by 4 cycles of freeze/thaw with liquid nitrogen. Efficient lysis was assessed by light microscopy. Debris and intact parasites were pelleted by centrifugation at 10,000 g for 10 min at 4°C. Soluble fractions were incubated overnight at 4°C with 20 μl of the antibody of interest. The next day, 100 μl of Sepharose bound Protein A or Protein G (Santa Cruz) for rabbit or mouse antibody, respectively, were added and incubated at room temperature for 2 h. Complexes were washed six times with Co-IP wash buffer (50 mM Tris pH 8, 200 mM NaCl, 2 mM EDTA, 1% NP-40) supplemented with protease inhibitor, then resuspended in 200 μl of SDS-PAGE loading buffer and boiled for 5 min. Elution fractions were used either for mass spectrometry or western blotting. Negative controls were performed using the pre-immune serum for each antibody or ProteinA/G Sepharose alone. Four independently obtained samples were analyzed. Sample 1 consisted of proteins obtained from a wild type parasite strain using rabbit serum raised against TgSMC1. Sample 2 was obtained using the same a-TgSMC1 antibody but subjected to affinity purification prior to the experiment. Sample 3 consisted of proteins obtained from the TgSMC1-YFP strain using a-GFP and Sample 4 was obtained from a wild type strain using a-GFP, and served as a negative control. **Figure 4A** shows sample 3 as a representative example of the immunoprecipitation scheme.

### Construction of Tagged Reporter Parasites

Toxoplasma gondii RH strain parasites were maintained by serial passage in human foreskin fibroblast (HFF) cells and genetically manipulated as previously described (Jacot et al., 2013). To tag the genomic locus of TgSMC1, TgExportin1, TgImportin1, and TgSUN1 with a 3xHA or a YFP tag, ~1,500 bp of the open reading frame ending before the stop codon were amplified from *T. gondii* genomic DNA. All primer sequences used are shown in Table S2. These amplicons were cloned via ligation independent cloning (LIC) (Aslanidis and de Jong, 1990) into the pLIC-HA-CAT or pLIC-YFP-DHFR vector, respectively, to create in-frame fusions (Huynh and Carruthers, 2009). Transgenic clones were established by transfection of ΔKu80-TaTi parasites and chloramphenicol or pyrimethamine selection, respectively. Integration was confirmed by PCR and western blot in all cases.

### Protein Expression and Antibody Production

Sequences encoding for the last 400 C-terminal amino-acids of TgSMC1 were amplified from *T. gondii* cDNA and inserted into plasmid pAVA-421 6xHis (Alexandrov et al., 2004). Recombinant fusion protein was purified on Ni^2^-NTA resin (Qiagen, Hilden, Germany). Rabbits were immunized with 1 mg of purified protein, and serum was collected after 10 weeks (Cocalico Biologicals, Reamstown, PA, USA). Mice were immunized with 0.4 mg of purified protein, and serum was collected after 3 weeks.

### Fluorescence Microscopy

For immunofluorescence assays, host cells (HFF) were inoculated onto coverslips and infected with parasites. Coverslips were fixed 24 h after infection and processed as previously described (Francia et al., 2011). Primary antibodies used were mouse anti-alpha tubulin at a dilution of 1:1,000 (12G10, a gift of Jacek Gaertig, University of Georgia), rabbit anti-Centrin1 at 1:1,000 (gift of Iain Cheeseman, MIT), mouse anti-GFP at 1:1,000–1:400 (Torry Pines Biolabs), rat anti-HA at 1:1,000 (clone 3F10, Roche), mouse anti-IMC1 mAb 45.15 at 1:1,000 (gift of Gary Ward, University of Vermont), mouse anti-TgChromo1 at 1:1,000 (Gissot et al., 2012), mouse anti-CenH3 (Francia et al., 2012) at 1:20, rabbit anti-MORN1 (Gubbels et al., 2006) at 1:250, and rabbit and mouse anti-SMC1 at 1:1,000 (this study). The secondary antibodies used were AlexaFluor 350, AlexaFluor 488, and AlexaFluor 546 (Invitrogen), at a dilution of 1:2,000. Images were collected on an Applied Precision Delta Vision inverted epifluorescence microscope using a UPlans APO 100×/1.40 oil lens. Images were subjected to deconvolution and contrast adjustment using Applied Precision software (Softworx). For quantitative image analysis (as described in the results section) a minimum of 50 vacuoles were scored in at least three independent experiments. Super-Resolution images were acquired using the Zeiss ELYRA S1 (SR-SIM) microscope. Images were collected and processed using Zeiss Zen software. Means and standard deviations were calculated and plotted using Graph Pad Prism Version 5.0c (La Jolla, California, USA).

### Transmission Electron Microscopy

Infected cells were fixed in 2% glutaraldehyde in sodium phosphate buffer 0.1 M, pH 7.4, followed by post-fixation with 1% osmium tetroxide in sodium phosphate buffer, alcohol dehydration, and Epon resin embedding. Serial sections were obtained with a Leica UCT cryo-ultramicrotome, collected in carbon coated single hole grids and observed in a JEOL 1200 EX transmission electron microscope.

### Western Blotting

Western blotting was performed as previously described (Brooks et al., 2011). We used anti-HA (Roche) antibodies at a dilution of 1:1,000, anti-tubulin at 1:1,000, anti-GFP at 1:500, anti-CenH3 at 1:500 and anti-SMC1 antibodies at a dilution of 1:1,000. Pre-immune sera for anti-SMC1 antibodies were used at a comparable dilution. Horseradish peroxidase (HRP)-conjugated anti-rat, anti-mouse, or anti-rabbit antibody (Pierce) were used at a dilution of 1:20,000

## Results

To investigate the mechanism mediating centromere clustering in *T. gondii* we propose to test two alternative hypotheses. First, we envision that a persistent microtubules spindle could constitutively interact with centromeres, thus maintaining their position, and ascribing the centrosome (MTOC) direct involvement in the process (Figure 1B). Alternatively, proteins present at the centromere mediate the interaction between it and the nuclear envelope (Figure 1B).

We first set out to investigate whether microtubules mediate centromere clustering. To test this, we subjected parasites to treatment with oryzalin, a tubulin-binding drug which prevents tubulin polymerization in Apicomplexa (Stokkermans et al., 1996; Morrissette et al., 2004). At concentrations of 2.5 mM oryzalin prevents polymerization of microtubules into daughter cells as well as the mitotic spindle (Stokkermans et al., 1996). Parasites expressing TgCenH3-HA (Brooks et al., 2011), a marker for centromeres, were subjected to treatment with 2.5 mM oryzalin for 24 h, fixed and observed by immunofluorescence assay (IFA) staining for a-HA and a-IMC1. IMC1 (Inner membrane complex protein 1) marks of the outline of dividing and non-dividing parasites. In dividing parasites, IMC1 labels the emerging daughter cell structures (Figure 1C). Upon drug treatment, parasites continue to grow and replicate their DNA but fail to assemble daughter cells (Figure 1C). Interphase as well as dividing parasites treated with oryzalin exhibit continued clustered localization for TgCenH3 (Figures 1B,C). These results suggested that the mitotic spindle is likely not responsible for centromere clustering during interphase. However, consistent with previous reports that oryzalin disrupts nuclear division (Morrissette and Sibley, 2002b), we note that oryzalin-treated parasites frequently fail to segregate their genome properly (Figure 1C). However, we cannot rule out incomplete spindle disruption upon drug treatment.

To independently examine interphase nuclei in *T. gondii*, we serially sectioned fixed parasites and observed them by transmission electron microscopy (Figures 1D,E). In all cases, the entire nucleus was sectioned and in most sets sections spanned the entire parasite. Parasites were assigned to interphase by the presence of a single, unduplicated centrosome, and the absence of budding daughters. Upon three dimensional reconstruction, we observed that while spindle microtubules are readily observed in dividing parasites (duplicated centrosomes) (Figure 1E) they cannot be detected in interphase parasites (Figure 1D). Overall, we could detect intranuclear microtubules in 98% of the dividing parasites (*n* = 13), while microtubules were seen only in 4% of nuclei considered to be in interphase by our morphological criteria (*n* = 60) (Figure 1F). The latter may represent parasites just emerged from mitosis.

Local actin polymerization was reported to affect telomere positioning in the *P. falciparum* nucleus (Zhang et al., 2011). To assess a potential role of actin in centromere clustering, parasites were treated with Cytochalasin D, an actin de-polymerizing agent. Treated parasites did not exhibit centromere dispersion (Figure S1B). Similarly, *a T. gondii* temperature sensitive mutant of the nuclear actin ARP4 exhibits normal centromere clustering at the restrictive temperature (Figure S1D) (Suvorova et al., 2012). Taken together, our pharmacological, ultra-structural and genetic analysis, strongly suggest that neither microtubules nor actin filaments are responsible for persistent centromere clustering in interphase.

We next set out to identify chromatin-binding factors which could potentially mediate centromere clustering by *in silico* identification of known centromeric proteins. Structural Maintenance of Chromosome proteins (SMCs) are a family of ATPases with multiple roles in chromatin organization during mitosis and meiosis (Jeppsson et al., 2014; Uhlmann, 2016). Homologs of Structural Maintenance of Chromosomes 1 (SMC1) have been implicated in the control of gene expression, DNA repair and recombination, cross linking of mitotic spindle microtubules and membrane anchoring of heterochromatin (Nasmyth and Haering, 2009; Wong, 2010). Importantly, the yeast and *Drosophila* SMC1s have been shown to directly associate with the centromeric histone H3 (Nasmyth and Haering, 2009; Wong, 2010). Searching for homologs of the *Saccharomyces cerevisiae* SMCs we identified four candidate genes for SMC proteins in the *T. gondii* genome (Figure S2A). Maximum likelihood phylogenetic tree of the full length protein coding sequences showed that each of *T. gondii*'s predicted SMC protein coding genes clustered with a given SMCs sub-class.TgME49_288700 clusters with SMC1-like SMCs; TgME49_297800 is more closely related to SMC2 from yeast and plants, while TgME49_106310 and TgME49_231170 are homologous to SMC3 and SMC4, respectively (Figure S2A).

**Figure 2:**
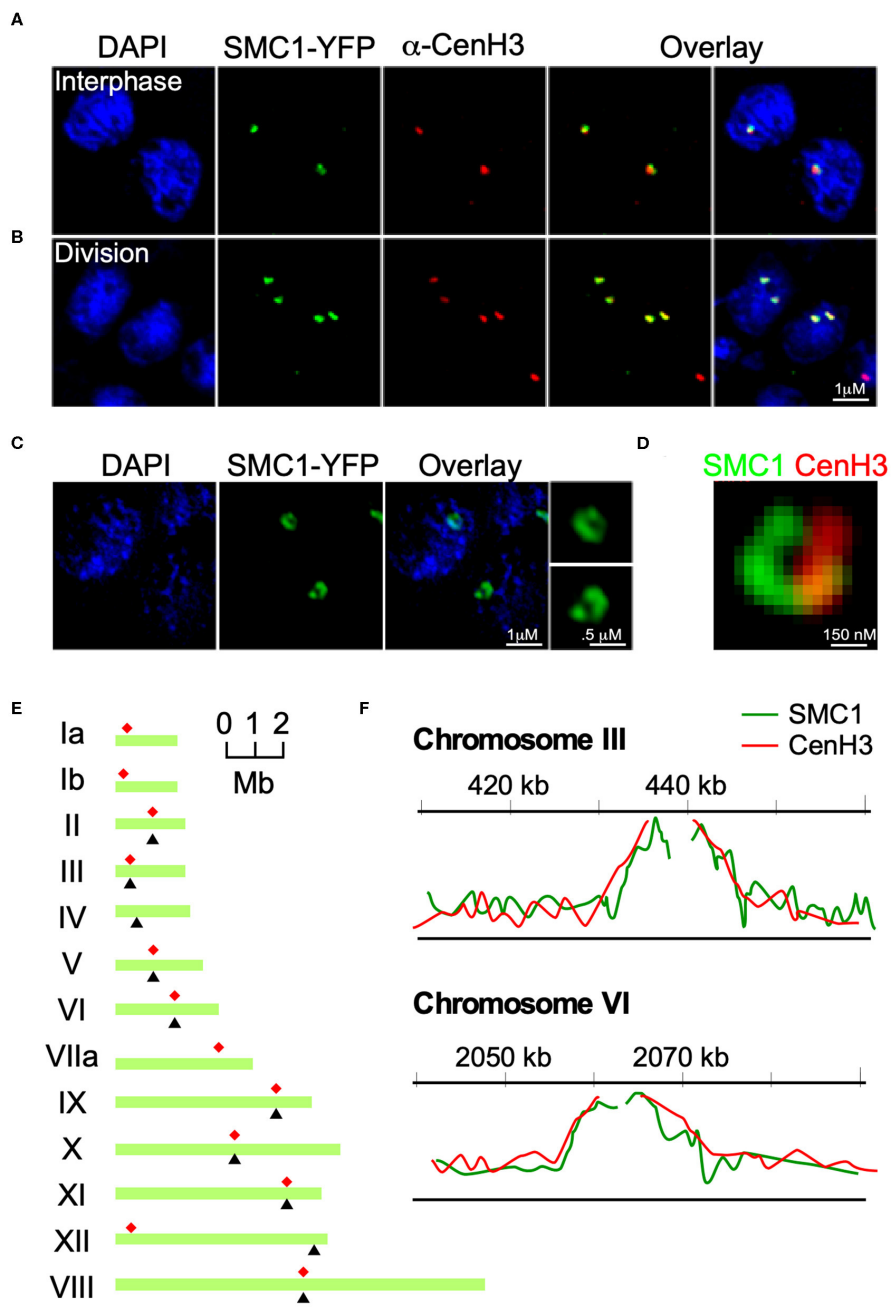
TgSMC1 is a persistent centromeric protein. **(A,B)** Immunofluorescence assay. TgSMC1-YFP (green) was co-stained with anti-TgCenH3 antibodies (green). The signals for TgSMC1 and the marker for the *T. gondii* centromeres show tight co-localization both in interphase **(A)** and dividing **(B)** parasites. TgSMC1 persists at the centromere both during all stages of mitosis and interphase. **(C)** Super Resolution (SR-SIM) image of TgSMC1-YFP stained with anti-GFP. TgSMC1 localization appears to be in the shape of a hollow oval **(D)** Zoom of the SR-SIM image of TgSMC1-YFP, co-stained with anti-GFP and anti-TgCenH3. TgSMC1 (green) appears to surround the centromeres marked by TgCenH3 (red). **(E)** Schematic representation of *T. gondii's* chromosomes. Red asterisks indicate the position of the centromeres, mapped previously, for each chromosome. Black arrowheads correspond to the hybridization peaks obtained from the TgSMC1-HA cell line ChIP-CHIP experiments for each chromosome. **(F)** Hybridization peaks on a microarray CHIP covering the genome of *T. gondii*, of immunoprecipitated chromatin from the TgSMC1-HA cell line (green line). Chromosomes III and VI are shown as representative examples. Our previous ChIP-CHIP results using the TgCenH3-HA cell line (red line) are shown, overlaid, as reference.

To further investigate the SMC1 homolog in *T. gondii* we generated strains with an insertion of a triple HA tag or a yellow fluorescent protein (YFP) cassette at the 3′ end of TgME49_288700 (from here on referred to as TgSMC1, Figure S2B). In addition, we raised mouse and rabbit anti-sera against a recombinant C-terminal fragment of TgSMC1 consisting of the 400 C-termini amino-acids of the protein. These anti-sera recognize a single protein of a size consistent with the predicted molecular mass of 183 kD (or 211 for the YFP fusion protein, respectively, Figure S2C). Using these reagents we investigated the localization of TgSMC1 by IFA. When co-stained with a monoclonal antibody raised against TgCenH3 (Brooks et al., 2011) we observed that TgSMC1 nuclear punctae coincide with TgCENH3 both in interphase and in dividing parasites (Figures 2A,B). Interestingly, when observed by structured Illumination super resolution microscopy (SIM-SR), the localization of TgSMC1 is better defined as a semi-circle arranged around the spot filled by the centromeres marked by TgCenH3 (Figures 2C,D).

To unequivocally determine whether TgSMC1 is a centromeric protein, we immunoprecipitated TgSMC1-associated chromatin, and probed a microarray chip covering most of the *T. gondii* genome with the precipitated DNA (ChIP-CHIP). Significant hybridization was obtained for 10 chromosomes. The hybridization peaks for chromosomes II, III, V, VI, and VIII-XI coincide with the position of the centromere on these chromosomes as mapped by ChIP-CHIP of TgCenH3 (Figure 2E). Moreover, TgSMC1 ChIP-CHIP hybridization signal shows almost perfect overlap with the chromatin regions bound by TgCENH3 (Figure 2F). Taken together, TgSMC1 localization appears intimately linked to the centromere.

**Figure 3:**
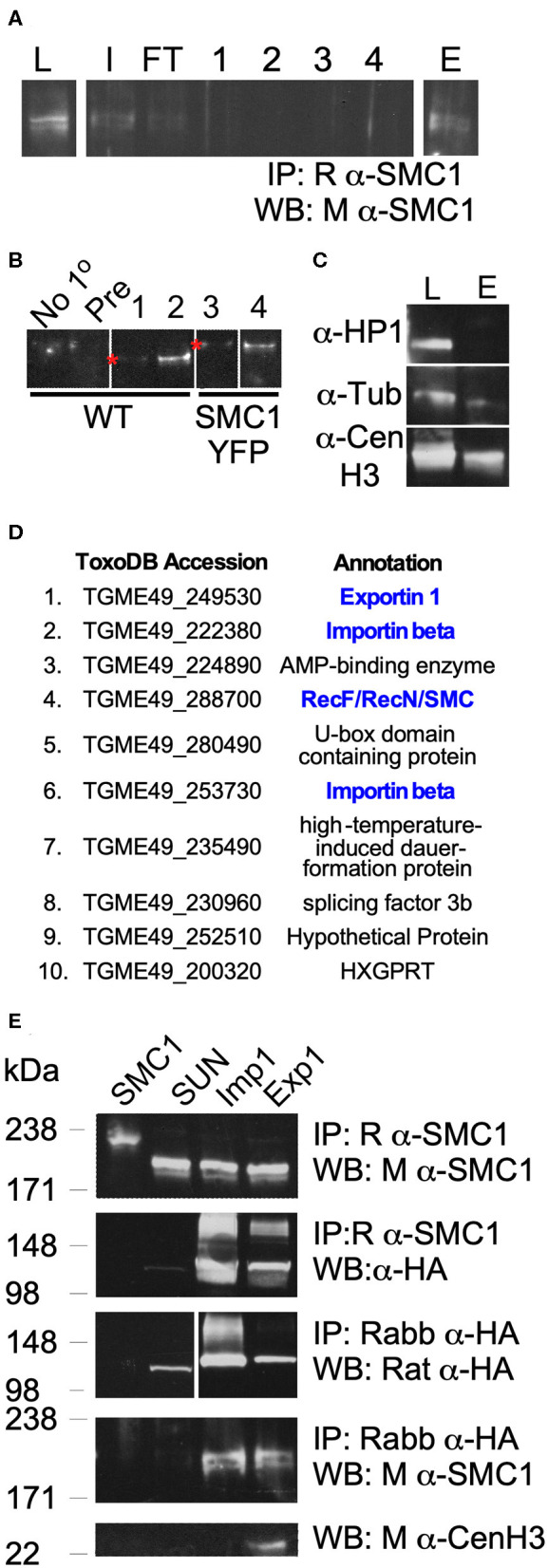
TgSMC1 Co-precipitates with Peripheral Components of the Nuclear Pore Complex. **(A)** Representative western blot of an immuno-precipitation experiment from parasite lysate using an anti-TgSMC1 antibody (L, lysate; I, input; FT, flow through; 1–4, washes; E, elution). **(B)** Western Blot. Elutions fractions E of immunoprecipitations using no primary antibody (No-1°), pre-immune sera (Pre) or the following antibodies: 1- Rabbit anti-SMC1, 2- Affinity Purified Rabbit anti-SMC1, 3, and 4- anti-GFP, and probed with mouse anti-SMC1. The strains used to generate parasite lysates (input) are specified below. **(C)** Western blot. Parasite lysate or the elution fraction of an immuno-precipitation using rabbit anti-SMC1 were probed with the indicated antibodies. Mouse anti-HP1 recognizes TgChromo1, a chromodomain protein which binds peri-centromeric DNA, and was used as a control for the specificity of our pull downs (L, total lysate; E, elution fraction). **(D)** The 10 most abundant co-precipitants of TgSMC1 are listed. Complete results of the Mass spectrometry analysis of all elution fractions of multiple immuno-precipitation experiments can be found in Table S1. Proteins highlighted in blue were followed up. **(E)** TgExp1 and TgImp1 (TgME49_249530 and TgME49_253730 respectively), which co-immunoprecipitated with TgSMC1, were endogenously tagged with a C-terminal 3xHA. TgSUN1 (TgME49_288530) is annotated as a hypothetical protein containing a Sun domain. This protein was represented by 2 peptides in the Mass spectrometry analysis of SMC1-YFP anti-GFP Co-IP, but was not found in other samples, and was used as a negative control (Table S1). TgSMC1 was pulled down using Rabbit anti-SMC1 antibodies in TgSMC1-YFP, TgImp1-HA, TgExp1-HA, and TgSUN-HA cells lines, and probed with mouse anti-SMC1 or anti-HA. Conversely, TgImp1, TgExp1, and TgSUN were pulled down using anti-HA antibodies, and the elution fractions were probed with anti-TgSMC1. These results recapitulate our LC-MS results. In addition to co-precipitating with TgSMC1, TgExp1 also co-precipitates with TgCENH3.

Lastly, we determined potential interactors of TgSMC1 by co-immunoprecipitation (Figures 3A,B). Proteins co-immunoprecipitated with TgSMC1 were identified by LC-MS. TgSMC1's elution fraction contains a significant amount of TgCenH3, suggesting that not only do they co-localize at the centromere but they also interact physically (Figure 3C). In contrast, TgChromo1 (Gissot et al., 2012) which binds chromatin immediately adjacent to the centromeres, does not co-precipitate with TgSMC1 (Figure 3C).

The ten most abundant proteins recovered in all four purifications are shown in Figure 3D. A complete list of LC-MS results can be found in Table S1 ordered by ascending order of accession number in the *T. gondii* genome database (Kissinger et al., 2003). Four protein-coding genes, one being TgSMC1, showed the highest number of unique peptides in all three positive samples, and a 10-fold enrichment in number of unique peptides as compared to the negative control. The remaining three are TgME49_222380, TgME49_253730, and TgME49_249530. The first two are annotated as proteins belonging to the Importinb family, while the third is annotated as Exportin 1. To further study TgSMC1's interactors, we generated reporter strains by introducing a 3-HA tag at the C-terminus of the proteins encoded by TgME49_253730 and TgME49_249530, which we named TgImportin1 (TgImp1) and TgExportin1 (TgExp1), respectively (Figure S3A). To validate these interactions, we performed reciprocal co-immunoprecipitation assays. As an internal control, we also generated a reporter strain for TgME49_288530 (TgSUN1) which presented 2 peptides in “sample 2” but was absent from all others. We were able to reproduce the co-precipitation of TgSMC1 with TgImp1 or TgExp1, but we did not detect interactions with TgSUN1 (Figure 3E). Interestingly, we found TgCenH3 to co-precipitate with TgExp1 (Figure 3E).

**Figure 4:**
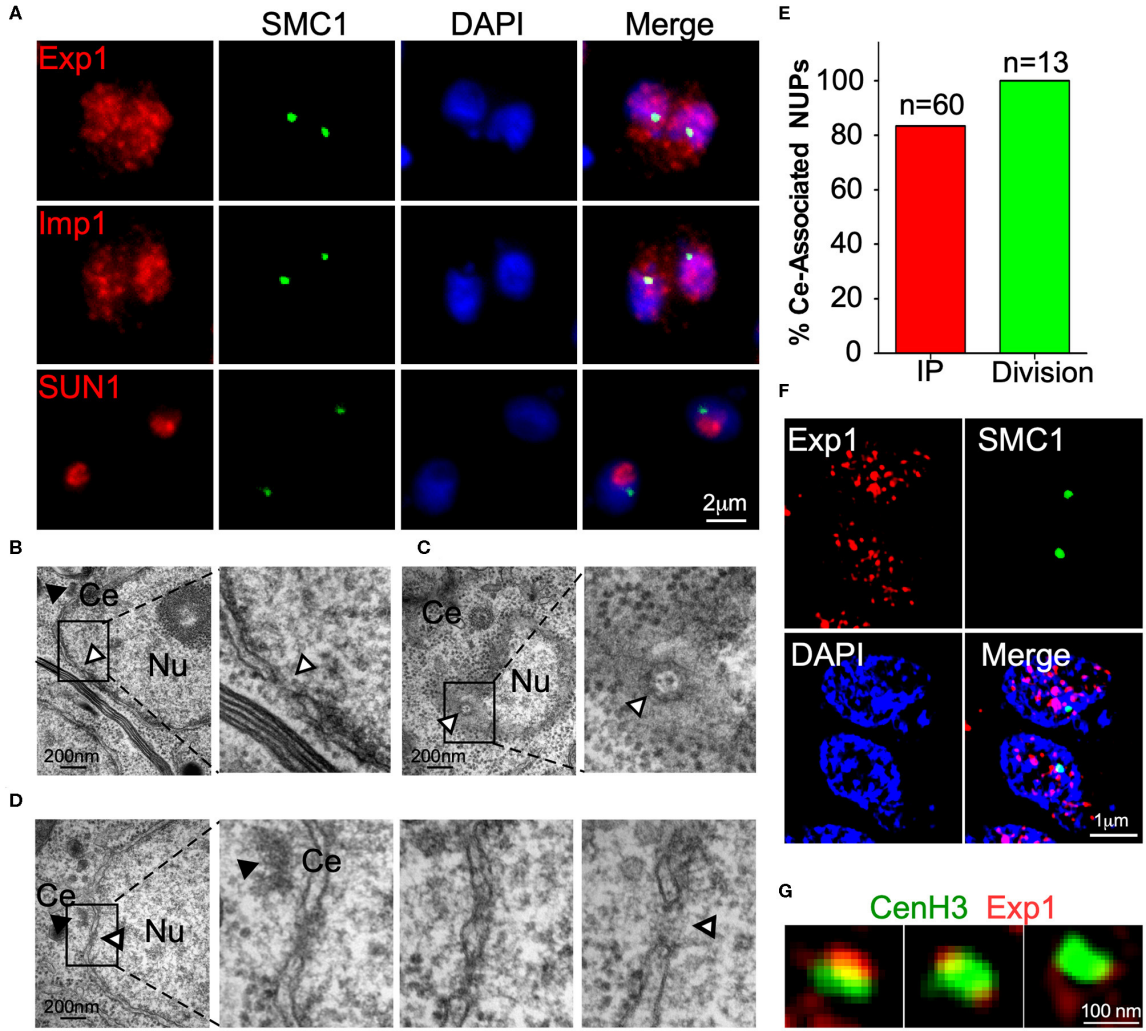
A nuclear pore is found in the proximity of the centrosome. **(A)** IFA of TgExp1-HA, TgImp –HA and TgSUN-HA using anti-HA antibodies reveal that these proteins localize to the nucleus. TgSUN1 is restricted to a discrete region of the nucleus, and does not co-localize with TgSMC1. **(B)** TEM section perpendicular to a nucleus (Nu). A nuclear pore (white arrowhead) is seen as a discontinuation in the nuclear envelope. **(C)** TEM section parallel to a nucleus (Nu). A nuclear pore (white arrowhead) is seen as a basket-like octagonal structure on the nuclear, distant from the centrosome (Ce, black arrowhead). **(D)** TEM Serial sections through an interphase nucleus reveal the presence of a nuclear pore (white arrowhead) visible as an interruption in the nuclear envelope adjacent to the centrosome (Ce, black arrowhead). Detailed panels are zoomed in at 200% **(E)**. The appearance of nuclear pores proximal to the centrosome (% Ce-associated NUPs) was quantified in serial sections of both dividing (green) and non-dividing (red) parasites. Note that the mitotic spindle penetrates and interrupts the nuclear envelope during division. Hence an interruption of the nuclear envelope, proximal to the centrosome, is observable in the vast majority of the dividing nuclei and is indistinguishable, by microscopy, from a canonical nuclear pore. **(F)** SR-SIM of TgExp1-HA reveals that TgExp1 localizes to heterogenous discrete foci in the nucleus. **(G)** TgExp1-HA foci co-localize with, flank or surround the localization of TgCenH3 marked by anti-TgCenH3 staining.

Importins and exportins are nuclear proteins which interact peripherally with transmembrane components of the nuclear pore complex (NPC). As expected, we determined that both TgExp1 and TgImp1 localize to the nucleus (Figure 4A). Super resolution microscopy revealed that TgExp1 localizes to discrete or clustered foci on the nucleus, consistent with its predicted NPC localization (Figure 4F). However, neither TgImp1 nor TgExp1 exclusively localize to the centromeric foci, consistent with their predicted peripheral localization to the NPC. We reasoned that if components of the NPC are involved in centromere clustering, this should be observable in sections of interphase nuclei in the vicinity of where centromeres cluster (i.e., the centrosome). Nuclear pores are readily observed by TEM in the *T. gondii* nucleus as interruptions in the nuclear envelope or as an oval with octagonal symmetry (Figures 4B,C). Indeed, when we observed the region of the nuclear envelope adjacent to the centrosome in interphase parasites sectioned perpendicularly, we could observe a pore in 84% of the nuclei (*n* = 60, Figures 4D,E). Co-labeling of either TgImp1 or TgExp1 and TgSMC1 revealed that these proteins co-localize (Figure 4F). Importantly, TgCenH3's localization coincides or is flanked by individual foci or clusters of TgExp1 (Figure 4G).

## Discussion

A long standing question in the field has been “how do apicomplexans keep track of the position of their chromosomes, without condensing their chromatin during division?” Chromosomes move, organize, and cluster by interacting with the mitotic spindle through kinetochore components that assemble at the centromere during mitosis. Electron microscopy studies in *T.gondii, Eimeria* spp., and *Sarcocystis neurona* demonstrated the presence of an intranuclear spindle (Dubremetz, 1973; Morrissette and Sibley, 2002a; Francia and Striepen, 2014). These studies identified spindle microtubules that link the centrosomes to what appear to be the kinetochores of the chromosomes (Dubremetz, 1973). More recently, *bona fide* residents of the mitotic spindle, such as the MT-binding protein TgEB1, have been identified suggesting a canonical mitotic spindle is assembled by Apicomplexa (Chen et al., 2015). Consistently, parasites treated with microtubule-disrupting agents fail to segregate their chromosomes properly (Morrissette and Sibley, 2002b), and knock-down of kinetochore proteins uncouple mitosis from cytokinesis (Farrell and Gubbels, 2014).

Our first set of experiments investigated whether cytoskeletal elements mediated centromere clustering. We demonstrated that neither microtubules of the mitotic spindle nor actin mediate this process. We propose that, instead, chromatin binding factors at the centromeres mediate the maintenance of their localization at the periphery of the nucleus. We identified and characterized the localization of a homolog of SMC1 in *Toxoplasma gondii*. SMCs are a family of proteins containing two ATPase globular domains at their C and N-terminals, and a hinge domain which establishes interactions with chromatin and other SMC and non-SMC proteins. SMCs have multiple roles in higher order chromatin organization and dynamics, powered by the hydrolysis of ATP (Losada and Hirano, 2005; Hirano, 2006). We determined that TgSMC1 is a centromere-associated protein which interacts with the centromeric histone variant H3, TgCenH3, and centromeric chromatin. SMC1 interactions with CenH3 homologs have been previously reported in yeast and *Drosophila* (Tanaka et al., 1999; Losada and Hirano, 2000).

SMC1 homologs have a role in chromosome segregation during mitosis as part of the cohesin complex, which ensures the maintenance of sister chromatid cohesion until chromosomes separate in anaphase (Onn et al., 2008). Typically, SMC1 localizes to sister chromatids, in the proximities of or at the centromeres, during mitosis and up until late metaphase/early anaphase, but it is absent from centromeric regions outside of mitosis (Gruber et al., 2003; Huang et al., 2005; Peters et al., 2008). During interphase, SMC1 homologs normally localize to the cytosol or associate with non-centromeric chromatin (Losada et al., 1998; Losada and Hirano, 2000). In *Toxoplasma gondii*, however, we observed that TgSMC1 persists at the centromeres throughout the cell cycle. It is possible that it remains inactive at the centromeres outside of mitosis, and that its activity depends on interacting partners or specific activation during mitosis. Alternatively, TgSMC1 could play additional roles in chromatin organization in *T. gondii*. Cohesin has been shown to contribute to gene regulation, DNA damage repair, transcriptional control, and maintenance of higher order chromatin structure in other systems (Peters et al., 2008). In human cells, SMC1 has been shown to mediate transcriptional insulation by binding chromatin boundaries in post-mitotic cells (Parelho et al., 2008; Peric-Hupkes and van Steensel, 2008; Wendt et al., 2008). Interestingly, in the closely related Apicomplexa *Eimeria tenella*, SMC1 is part of a plaque formed at the nuclear envelope to which telomeres attach during meiotic division (del Cacho et al., 2010). Our results suggest that TgSMC1 could fulfill a similar role in mediating the attachment of centromeres to the nuclear envelope.

The latter is supported by our identification of TgSMC1 interactors. In particular, we determined that TgSMC1 co-precipitated with soluble proteins predicted to function at nuclear pores; TgExportin7, TgExportin1, and TgImportin1. Nuclear pores are basket-like structure of octagonal shape and consist of a central scaffold which spans the nuclear envelope (Hoelz et al., 2011; Kahms et al., 2011). Proteins of the nuclear pore are collectively known as nucleoporins (NUPs) are anchored by transmembrane domains, and form a molecular sieve by the presence of FG repeats in a central channel, preventing diffusion of molecules larger than 40 KDa or 5 nm. For larger molecules to travel through the pore, they must reversibly associate with FG nucleoporins (Wente and Rout, 2010; Hoelz et al., 2011). Translocation of large molecules depends on nuclear transport receptors (NTRs), i.e., importins, exportins, and transportins (Görlich and Kutay, 1999).

The TgSMC1 interactors identified, TgExp1 and TgImp1, localize to discrete foci in the nucleus, and co-localize or flank the location of centromeres in the nuclear periphery. By TEM we established that an opening in the nuclear envelope can be observed adjacent to the centrosome in interphase nuclei, implying that centromeres arrange in the vicinity of an NPC.

Proteins associated peripherally with the NPC, such as NTRs, have been shown to fulfill roles independent from their transport function. Several transportins have been shown to exert strong boundary activity by mediating the association of chromatin with core components of the nuclear pore. Intriguingly, like TgExp1 and TgImp1, they all belong to the Importin-β superfamily of NTRs. In particular Nup2, a peripheral NUP associated with the nuclear pore basket, is essential for boundary activity of transportins in yeast (Ishii et al., 2002; Shinkura and Forrester, 2002). *Schizosaccharomyces pombe*, a fission yeast which divides by closed mitosis and clusters centromeres, presents a TgExportin1 homolog, named CRM1, first identified in a cold-sensitive mutant screen (Adachi and Yanagida, 1989). Conspicuously, CRM1 has been shown to be essential for maintenance of centromere clustering during interphase (Adachi and Yanagida, 1989; Funabiki et al., 1993). Interestingly, characterization of the effect of temperature sensitive mutations identified that a point mutation caused CRM1 to mis-localize to the cytosol (Adachi and Yanagida, 1989), an effect that can be mimicked on wild type CRM1 by Leptomycin B treatment (Nishi et al., 1994; Kudo et al., 1999). At the restrictive temperature or upon Leptomycin B treatment, centromeres of *S. pombe* come apart and disperse in the nucleus.

Core nucleoporins (NUPs) have also been shown to directly interact with chromatin, and to regulate chromatin organization in other systems. Particularly relevant to this study; Nup98 co-precipitates with SMC1 in *Drosophila* (Wong and Blobel, 2008). ChIP-CHIP of Nup93 demonstrated direct chromatin association with the nuclear pore complex in human cells (Brown et al., 2008). Dynamic changes in the distribution of nuclear pores on the nuclear envelope were observed by elegant microscopy techniques during the intracellular development of the apicomplexan *Plasmodium falciparum* (Weiner et al., 2011). Late schizonts exhibit 2–6 nuclear pores per nucleus, which cluster together and invariably are surrounded by heterochromatin suggesting that nuclear pores associate with specific states of chromatin condensation (Weiner et al., 2011). Noteworthy, heterochromatin flanks the centromeres of *T. gondii* (Brooks et al., 2011; Gissot et al., 2012). A recent study which characterized the *T. gondii* Nup98 homolog (TgNUP302) revealed that this protein interacted with facilitates chromatin transcription complex (FACT) components, suggesting the existence of an NPC-chromatin interaction in *T. gondii* (Courjol et al., 2017). Therefore, centromere clustering could be part of a more general organizational scheme of nuclear elements in apicomplexan parasites, dependent on interactions with components of the nuclear envelope, and in particular with the NPC.

While we have started to unravel the mechanism by which centromeres are held in position at the nuclear envelope, we do not yet understand how centromeres are recruited to a specific site on the nuclear periphery, adjacent to the centrosome. Intriguingly, TgNUP302 was shown to physically associate to TGGT1_246190, a coiled-coiled protein (named TgCEP530) shown to localize at the centrosome, thereby identifying a physical connection between the nuclear pore complex and the centrosome (Courjol and Gissot, 2018). Together, the connection between nuclear pore components and the centrosome, and peripheral components of the nuclear pore-chromatin, described herein, could be the basis of the centromere-centrosome connection. Further study of centrosome-associated factors could shed light on the identity of components with roles in targeting or maintaining the position of centrosome-associated nuclear components.

## Data Availability Statement

The raw data supporting the conclusions of this article will be made available by the authors, without undue reservation, to any qualified researcher.

## Ethics Statement

The animal study was reviewed and approved by the University of Georgia Institutional Animal Care and Use Committee or IACUC.

## Author Contributions

MF designed and performed experiments and wrote the manuscript. L-MT, MC, and J-FD performed experiments. KK supervised L-MT and MC. BS secured funding, designed experiments, and wrote the manuscript. All authors contributed to the article and approved the submitted version.

## Conflict of Interest

The authors declare that the research was conducted in the absence of any commercial or financial relationships that could be construed as a potential conflict of interest.

## References

[B1] Adachi Y. Yanagida M. (1989). Higher order chromosome structure is affected by cold-sensitive mutations in a Schizosaccharomyces pombe gene crm1+ which encodes a 115-kD protein preferentially localized in the nucleus and its periphery. J. Cell Biol. 108, 1195–1207. 10.1083/jcb.108.4.1195 2647765 PMC2115495

[B2] Alexandrov A. Vignali M. LaCount D. J. Quartley E. de Vries C. De Rosa D.. (2004). A facile method for high-throughput co-expression of protein pairs. Mol. Cell. Proteomics 3, 934–938. 10.1074/mcp.T400008-MCP200 15240823

[B3] Aslanidis C. de Jong P. J. (1990). Ligation-independent cloning of PCR products (LIC-PCR). Nucleic Acids Res. 18, 6069–6074. 10.1093/nar/18.20.6069 2235490 PMC332407

[B4] Brooks C. F. Francia M. E. Gissot M. Croken M. M. Kim K. Striepen B. (2011). *Toxoplasma gondii* sequesters centromeres to a specific nuclear region throughout the cell cycle. Proc. Natl. Acad. Sci. U. S. A. 108, 3767–3772. 10.1073/pnas.1006741108 21321216 PMC3048097

[B5] Brown C. R. Kennedy C. J. Delmar V. A. Forbes D. J. Silver P. A. (2008). Global histone acetylation induces functional genomic reorganization at mammalian nuclear pore complexes. Genes Dev. 22, 627–639. 10.1101/gad.1632708 18316479 PMC2259032

[B6] Bunnik E. M. Venkat A. Shao J. McGovern K. E. Batugedara G. Worth D.. (2019). Comparative 3D genome organization in apicomplexan parasites. Proc. Natl. Acad. Sci. U.S.A. 116, 3183–3192. 10.1073/pnas.1810815116 30723152 PMC6386730

[B7] Chen C. T. Kelly M. De Leon J. Nwagbara B. Ebbert P. Ferguson D. J. P.. (2015). Compartmentalized Toxoplasma EB1 bundles spindle microtubules to secure accurate chromosome segregation. Mol. Biol. Cell 26, 4562–4576. 10.1091/mbc.E15-06-0437 26466679 PMC4678015

[B8] Courjol F. Gissot M. (2018). A coiled-coil protein is required for coordination of karyokinesis and cytokinesis in *Toxoplasma gondii*. Cell. Microbiol. 20:e12832. 10.1111/cmi.12832 29447426

[B9] Courjol F. Mouveaux T. Lesage K. Saliou J. M. Werkmeister E. Bonabaud M.. (2017). Characterization of a nuclear pore protein sheds light on the roles and composition of the *Toxoplasma gondii* nuclear pore complex. Cell. Mol. Life Sci. 74, 2107–2125. 10.1007/s00018-017-2459-3 28138739 PMC11107709

[B10] del Cacho E. Pagés M. Gallego M. Barbero J. L. Monteagudo L. Sánchez-Acedo C. (2010). Meiotic chromosome pairing and bouquet formation during Eimeria tenella sporulation. Int. J. Parasitol. 40, 453–462. 10.1016/j.ijpara.2009.09.008 19837073

[B11] Dubremetz J. F. (1973). Etude ultrastructurale de la mitose schizogonique chez la coccidie Eimeria necatrix (Johnson 1930). J. Ultrastruct. Res. 42, 354–376. 10.1016/S0022-5320(73)90063-4 4702924

[B12] Farrell M. Gubbels M. J. (2014). The Toxoplasma gondii kinetochore is required for centrosome association with the centrocone (spindle pole). Cell. Microbiol. 16, 78–94. 10.1111/cmi.12185 24015880 PMC3933516

[B13] Francia M. E. Jordan C. N. Patel J. D. Sheiner L. Demerly J. L. Fellows J. D.. (2012). Cell division in apicomplexan parasites is organized by a homolog of the striated rootlet fiber of algal flagella. PLoS Biol. 10:e1001444. 10.1371/journal.pbio.1001444 23239939 PMC3519896

[B14] Francia M. E. Striepen B. (2014). Cell division in apicomplexan parasites. Nat. Rev. Microbiol. 12, 125–136. 10.1038/nrmicro3184 24384598

[B15] Francia M. E. Wicher S. Pace D. A. Sullivan J. Moreno S. N. J. Arrizabalaga G. (2011). A Toxoplasma gondii protein with homology to intracellular type Na+/H+ exchangers is important for osmoregulation and invasion. Exp. Cell Res. 317, 1382–1396. 10.1016/j.yexcr.2011.03.020 21501607 PMC3096714

[B16] Funabiki H. Hagan I. Uzawa S. Yanagida M. (1993). Cell cycle-dependent specific positioning and clustering of centromeres and telomeres in fission yeast. J. Cell Biol. 121, 961–976. 10.1083/jcb.121.5.961 8388878 PMC2119680

[B17] Gissot M. Kelly K. A. Ajioka J. W. Greally J. M. Kim K. (2007). Epigenomic modifications predict active promoters and gene structure in *Toxoplasma gondii*. PLoS Pathog. 3:e77. 10.1371/journal.ppat.0030077 17559302 PMC1891328

[B18] Gissot M. Walker R. Delhaye S. Huot L. Hot D. Tomavo S. (2012). Toxoplasma gondii chromodomain protein 1 binds to heterochromatin and colocalises with centromeres and telomeres at the nuclear periphery. PLoS ONE 7:e32671. 10.1371/journal.pone.0032671 22427862 PMC3302879

[B19] Görlich D. Kutay U. (1999). Transport between the cell nucleus and the cytoplasm. Annu. Rev. Cell Dev. Biol. 15, 607–660. 10.1146/annurev.cellbio.15.1.607 10611974

[B20] Gruber S. Haering C. H. Nasmyth K. (2003). Chromosomal cohesin forms a ring. Cell 112, 765–777. 10.1016/S0092-8674(03)00162-4 12654244

[B21] Gubbels M. J. Vaishnava S. Boot N. Dubremetz J. F. Striepen B. (2006). A MORN-repeat protein is a dynamic component of the Toxoplasma gondii cell division apparatus. J. Cell Sci. 119, 2236–2245. 10.1242/jcs.02949 16684814

[B22] Gubbels M. J. White M. Szatanek T. (2008). The cell cycle and *Toxoplasma gondii* cell division: Tightly knit or loosely stitched? Int. J. Parasitol. 38, 1343–1358. 10.1016/j.ijpara.2008.06.004 18703066

[B23] Hirano T. (2006). At the heart of the chromosome: SMC proteins in action. Nat. Rev. Mol. Cell Biol. 7, 311–322. 10.1038/nrm1909 16633335

[B24] Hoeijmakers W. A. M. Flueck C. Françoijs K. J. Smits A. H. Wetzel J. Volz J. C.. (2012). *Plasmodium falciparum* centromeres display a unique epigenetic makeup and cluster prior to and during schizogony. Cell. Microbiol. 14, 1391–1401. 10.1111/j.1462-5822.2012.01803.x 22507744

[B25] Hoelz A. Debler E. W. Blobel G. (2011). The structure of the nuclear pore complex. Annu. Rev. Biochem. 80, 613–643. 10.1146/annurev-biochem-060109-151030 21495847

[B26] Huang C. E. Milutinovich M. Koshland D. (2005). Rings, bracelet or snaps: Fashionable alternatives for Smc complexes. Philos. Trans. R. Soc. B Biol. Sci. 360, 537–542. 10.1098/rstb.2004.1609 15897179 PMC1569475

[B27] Huynh M.-H. Carruthers V. B. (2009). Tagging of endogenous genes in a *Toxoplasma gondii* strain lacking Ku80. Eukaryot. Cell 8, 530–539. 10.1128/EC.00358-08 19218426 PMC2669203

[B28] Ishii K. Arib G. Lin C. Van Houwe G. Laemmli U. K. (2002). Chromatin boundaries in budding yeast: the nuclear pore connection. Cell 109, 551–562. 10.1016/S0092-8674(02)00756-0 12062099

[B29] Jacot D. Meissner M. Sheiner L. Soldati-Favre D. Striepen B. (2013). “Genetic manipulation of *Toxoplasma gondii*,” in Toxoplasma Gondii: The Model Apicomplexan - Perspectives and Methods: Second Edition, eds L. M. Weiss and K. Kim (Burlington, VT: Elsevier Academic Press, 577–611.

[B30] Jeppsson K. Kanno T. Shirahige K. Sjögren C. (2014). The maintenance of chromosome structure: positioning and functioning of SMC complexes. Nat. Rev. Mol. Cell Biol. 15, 601–614. 10.1038/nrm3857 25145851

[B31] Kahms M. Hüve J. Wesselmann R. Farr J. C. Baumgärtel V. Peters R. (2011). Lighting up the nuclear pore complex. Eur. J. Cell Biol. 90, 751–758. 10.1016/j.ejcb.2011.04.004 21632146

[B32] Kissinger J. C. Gajria B. Li L. Paulsen I. T. Roos D. S. (2003). ToxoDB: accessing the *Toxoplasma gondii* genome. Nucleic Acids Res. 31, 234–236. 10.1093/nar/gkg072 12519989 PMC165519

[B33] Kudo N. Matsumori N. Taoka H. Fujiwara D. Schreiner E. P. Wolff B.. (1999). Leptomycin B inactivates CRM1/exportin 1 by covalent modification at a cysteine residue in the central conserved region. Proc. Natl. Acad. Sci. U.S.A. 96, 9112–9117. 10.1073/pnas.96.16.9112 10430904 PMC17741

[B34] Losada A. Hirano M. Hirano T. (1998). Identification of Xenopus SMC protein complexes required for sister chromatid cohesion. Genes Dev. 12, 1986–1997. 10.1101/gad.12.13.1986 9649503 PMC316973

[B35] Losada A. Hirano T. (2000). New light on sticky sisters. Curr. Biol. 10:R615. 10.1016/S0960-9822(00)00670-9 10996082

[B36] Losada A. Hirano T. (2005). Dynamic molecular linkers of the genome: the first decade of SMC proteins. Genes Dev. 19, 1269–1287. 10.1101/gad.1320505 15937217

[B37] Morrissette N. S. Mitra A. Sept D. Sibley L. D. (2004). Dinitroanilines bind α-tubulin to disrupt microtubules. Mol. Biol. Cell 15, 1960–1968. 10.1091/mbc.e03-07-0530 14742718 PMC379290

[B38] Morrissette N. S. Sibley L. D. (2002a). Cytoskeleton of apicomplexan parasites. Microbiol. Mol. Biol. Rev. 66, 21–38. 10.1128/MMBR.66.1.21-38.2002 11875126 PMC120781

[B39] Morrissette N. S. Sibley L. D. (2002b). Disruption of microtubules uncouples budding and nuclear division in *Toxoplasma gondii*. J. Cell Sci. 115, 1017–1025. 11870220 10.1242/jcs.115.5.1017

[B40] Nasmyth K. Haering C. H. (2009). Cohesin: its roles and mechanisms. Annu. Rev. Genet. 43, 525–558. 10.1146/annurev-genet-102108-134233 19886810

[B41] Nishi K. Yoshida M. Fujiwara D. Nishikawa M. Horinouchi S. Beppu T. (1994). Leptomycin B targets a regulatory cascade of crm1, a fission yeast nuclear protein, involved in control of higher order chromosome structure and gene expression. J. Biol. Chem. 269, 6320–6324. 8119981

[B42] Onn I. Heidinger-Pauli J. M. Guacci V. Ünal E. Koshland D. E. (2008). Sister chromatid cohesion: a simple concept with a complex reality. Annu. Rev. Cell Dev. Biol. 24, 105–129. 10.1146/annurev.cellbio.24.110707.175350 18616427

[B43] Parelho V. Hadjur S. Spivakov M. Leleu M. Sauer S. Gregson H. C.. (2008). Cohesins functionally associate with CTCF on mammalian chromosome arms. Cell 132, 422–433. 10.1016/j.cell.2008.01.011 18237772

[B44] Peric-Hupkes D. van Steensel B. (2008). Linking cohesin to gene regulation. Cell 132, 925–928. 10.1016/j.cell.2008.03.001 18358805

[B45] Peters J. M. Tedeschi A. Schmitz J. (2008). The cohesin complex and its roles in chromosome biology. Genes Dev. 22, 3089–3114. 10.1101/gad.1724308 19056890

[B46] Shinkura N. Forrester W. C. (2002). Pushing the envelope: chromatin boundaries at the nuclear pore. Mol. Cell 9, 1156–1158. 10.1016/S1097-2765(02)00556-7 12086612

[B47] Stokkermans T. J. W. Schwartzman J. D. Keenan K. Morrissette N. S. Tilney L. G. Roos D. S. (1996). Inhibition of *Toxoplasma gondii* replication by dinitroaniline herbicides. Exp. Parasitol. 84, 355–370. 10.1006/expr.1996.0124 8948325

[B48] Suvorova E. S. Lehmann M. M. Kratzer S. White M. W. (2012). Nuclear actin-related protein is required for chromosome segregation in *Toxoplasma gondii*. Mol. Biochem. Parasitol. 181, 7–16. 10.1016/j.molbiopara.2011.09.006 21963440 PMC3767130

[B49] Tanaka T. Cosma M. P. Wirth K. Nasmyth K. (1999). Identification of cohesin association sites at centromeres and along chromosome arms. Cell 98, 847–858. 10.1016/S0092-86740081518-4 10499801

[B50] Uhlmann F. (2016). SMC complexes: from DNA to chromosomes. Nat. Rev. Mol. Cell Biol. 17, 399–412. 10.1038/nrm.2016.30 27075410

[B51] Weiner A. Dahan-Pasternak N. Shimoni E. Shinder V. von Huth P. Elbaum M.. (2011). 3D nuclear architecture reveals coupled cell cycle dynamics of chromatin and nuclear pores in the malaria parasite *Plasmodium falciparum*. Cell. Microbiol. 13, 967–977. 10.1111/j.1462-5822.2011.01592.x 21501361

[B52] Wells J. Farnham P. J. (2002). Characterizing transcription factor binding sites using formaldehyde crosslinking and immunoprecipitation. Methods 26, 48–56. 10.1016/S1046-20230200007-5 12054904

[B53] Wendt K. S. Yoshida K. Itoh T. Bando M. Koch B. Schirghuber E.. (2008). Cohesin mediates transcriptional insulation by CCCTC-binding factor. Nature 451, 796–801. 10.1038/nature06634 18235444

[B54] Wente S. R. Rout M. P. (2010). The nuclear pore complex and nuclear transport. Cold Spring Harb. Perspect. Biol. 2, a000562 10.1101/cshperspect.a000562 20630994 PMC2944363

[B55] White M. W. Suvorova E. S. (2018). Apicomplexa cell cycles: something old, borrowed, lost, and new. Trends Parasitol. 34, 759–771. 10.1016/j.pt.2018.07.006 30078701 PMC6157590

[B56] Wong R. W. (2010). An update on cohesin function as a “molecular glue” on chromosomes and spindles. Cell Cycle 9, 1754–1758. 10.4161/cc.9.9.11806 20436296

[B57] Wong R. W. Blobel G. (2008). Cohesin subunit SMC1 associates with mitotic microtubules at the spindle pole. Proc. Natl. Acad. Sci. U.S.A. 105, 15441–15445. 10.1073/pnas.0807660105 18832153 PMC2557025

[B58] Zhang Q. Huang Y. Zhang Y. Fang X. Claes A. Duchateau M.. (2011). A critical role of perinuclear filamentous Actin in spatial repositioning and mutually exclusive expression of virulence genes in malaria parasites. Cell Host Microbe 10, 451–463. 10.1016/j.chom.2011.09.013 22100161 PMC7116676

