# Supplementary material for: A Homolog of Structural Maintenance of Chromosome 1 Is a Persistent Centromeric Protein Which Associates With Nuclear Pore Components in Toxoplasma gondii
Source: Front Cell Infect Microbiol. 2020 Jul 2;10:295. doi: 10.3389/fcimb.2020.00295 (PMC7343853; doi:10.3389/fcimb.2020.00295)
Supplement: Supplementary file 1 [file Data_Sheet_1.docx]

**
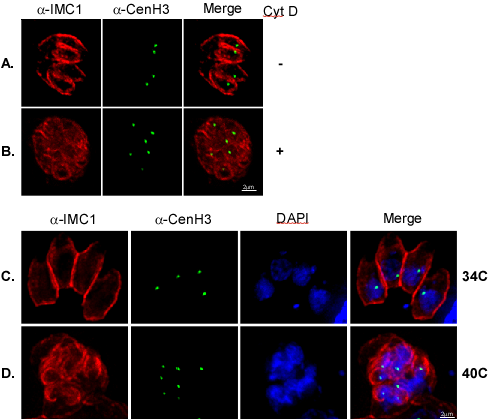
**

**Figure S1. Actin is not involved in centromere clustering**. Immunofluorescence assay of DMSO (**A**) (control) or Cytochalasin D (**B**) treated parasites with anti-IMC1 to label the parasites’ outline and anti-TgCenH3 to label the centromeres. Centromeres remain clustered as one or two foci per parasite upon treatment with Cytochalasin D. **C.** and **D.** Immunofluorescence assay of a temperature sensitive mutant bearing a destabilizing mutation in a nuclear actin-like protein (ARP4). Centromeres, labeled with anti-TgCenH3, remain clustered both at the permissive (**C**), and restrictive (**D**) temperatures.


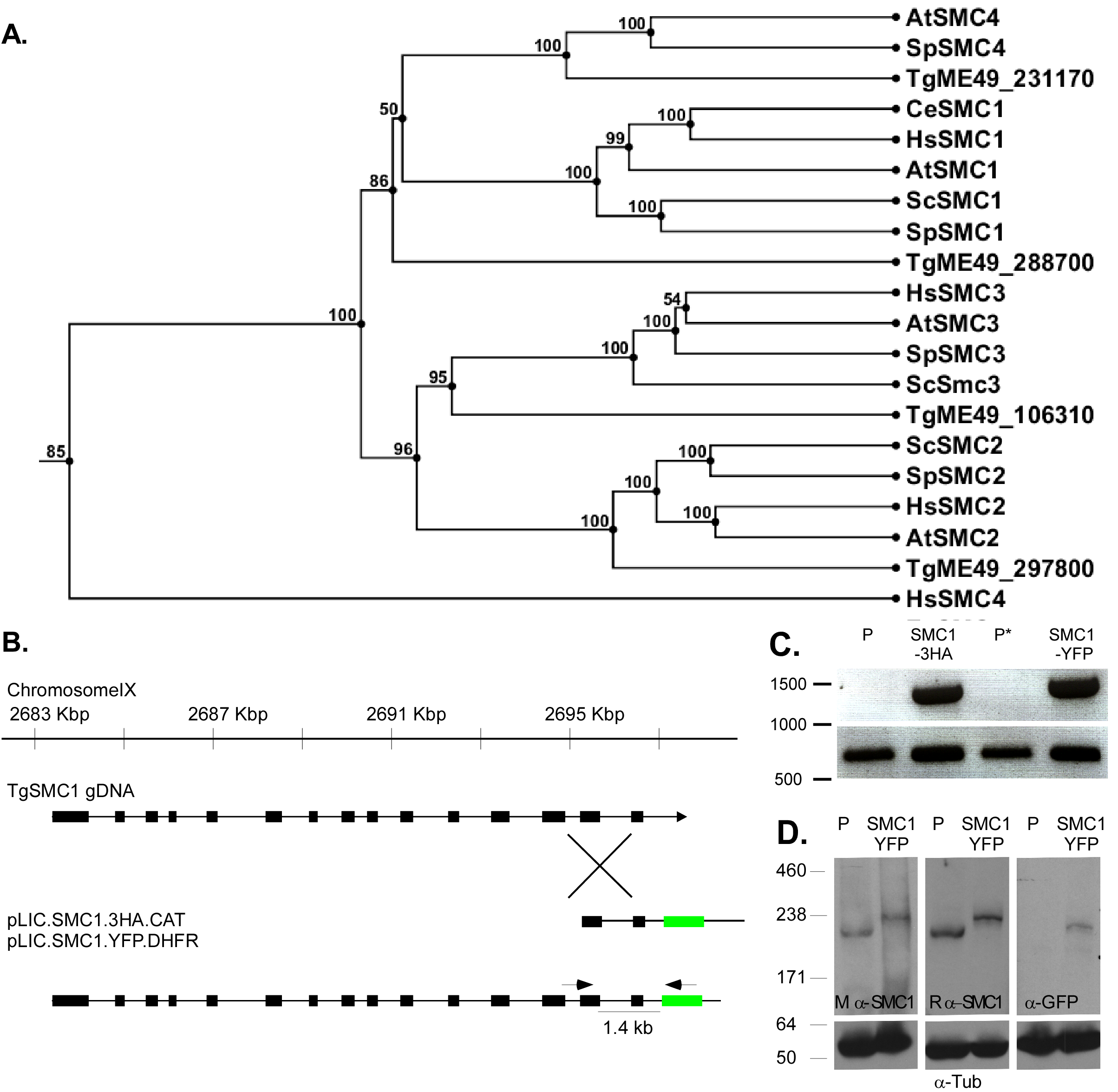


**Figure S2. Four Structural Maintenance of Chromosome (SMC) homologs are encoded in the *T. gondii* genome**. **A.** The genome of *T. gondii* encodes for four homologs of the Structural Maintenance of Chromosome (SMC) family of proteins, each clustering with a distinct functional clade. TgME49_297800 is an SMC1 homolog (TgSMC1). **B.** Two epitope tagged cell lines were generated by replacing the 3’ end of TgSMC1 with either a triple hemagglutinin (HA) or a yellow fluorescent protein (YFP). Arrows represent the approximate position of primers used to screen tagged clones generated from a parental wild type strain. **C.** PCR confirmation of tag insertion. The expected PCR product is detected in both tagged cell lines but not detected in parental cell lines. A control set of primers amplifies a PCR product from a different genomic location in all cell lines. **D.** Western blot shows that antibodies raised against the last 400 C-terminal amino-acids of TgSMC1 recognize the expected molecular weight protein in wild type parasites, as well as in a strain in which TgSMC1 was endogenously tagged with a C-terminal YFP (TgSMC1-YFP). Anti-GFP recognizes TgSMC1-YFP. Anti-tubulin was used as a control.


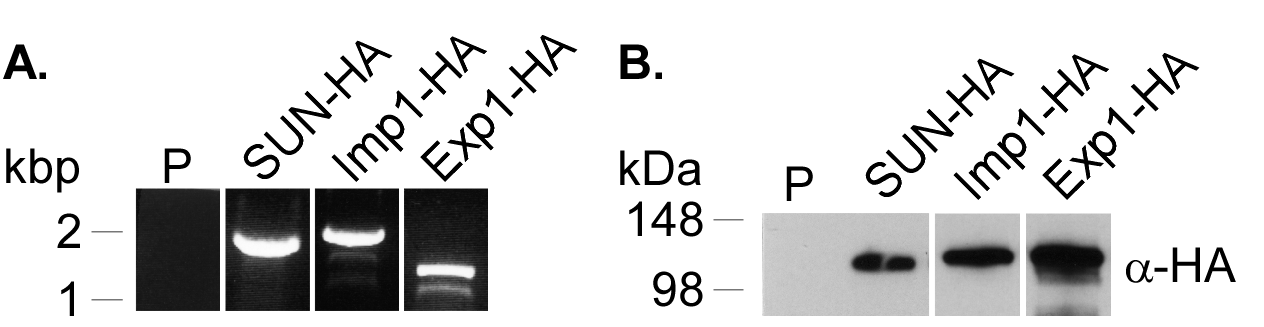


**Figure S3. Generation of epitope tagged TgSMC1-interactors.** Three epitope tagged cell lines (SUN-HA, Importin1-HA and Exportin1-HA) were generated by replacing the gene´s endogenous 3´end with a triple HA tag in the parental cell lines (P). **A.** PCR confirmation of tag insertion. Primers amplify the expected DNA band in tagged cell lines but not in the parental cell line. **B.** Western blot. Anti-HA antibody recognizes the expected molecular weight proteins in all three tagged cell lines.

**Table S1: Mass Spectrometry (LC-MS) Results of SMC1 Co-Immunoprecipitation**

|  |  |  |  |  | Sample**  (Unique Peptides) | | | |
| --- | --- | --- | --- | --- | --- | --- | --- | --- |
| ToxoDB Accession Number | Annotation | Av. Protein ID Prob. (%) | Mass (Da) | Av. Seq. Coverage (%) | α-SMC1 | α-SMC1* | α-GFP (SMC1-YFP) | (-) cont. |
| TGME49_249530 | exportin 1, putative | 100 | 129094 | 18 | 4 | 20 | 20 | 1 |
| TGME49_222380 | importin-beta N-terminal domain-containing protein | 100 | 129490 | 18 | 8 | 19 | 17 | 0 |
| TGME49_224890 | AMP-binding enzyme | 100 | 116710 | 9 | 5 | 14 | 3 | 0 |
| TGME49_288700 | RecF/RecN/SMC N terminal domain-containing protein | 100 | 183132 | 16 | 16 | 14 | 22 | 0 |
| TGME49_280490 | U-box domain-containing protein | 100 | 121998 | 8 | 4 | 13 | 7 | 0 |
| TGME49_253730 | importin-beta N-terminal domain-containing protein | 100 | 115148 | 12 | 2 | 10 | 9 | 0 |
| TGME49_235490 | High-temperature induced dauer formation protein homolog | 100 | 102120 | 12 | 4 | 8 | 6 | 0 |
| TGME49_230960 | splicing factor 3b, subunit 3, 130kD, putative | 99 | 135745 | 2.96 | 2 | 7 | 1 | 0 |
| TGME49_252510 | hypothetical protein | 100 | 80604 | 11 | 0 | 7 | 3 | 0 |
| TGME49_200320 | hypoxanthine-xanthine-guanine phosphoribosyl transferase (HXGPRT) | 100 | 31485 | 27 | 6 | 6 | 8 | 0 |
| TGME49_286080 | elongation factor 2 family protein | 100 | 113344 | 4.5 | 2 | 6 | 7 | 0 |
| TGME49_205220 | U5 snRNP-associated subunit, putative | 75 | 122776 | 3.42 | 0 | 5 | 1 | 0 |
| TGME49_244110 | nucleosome assembly protein (nap) protein | 100 | 48587 | 11 | 2 | 4 | 4 | 0 |
| TGME49_269990 | hypothetical protein | 100 | 216244 | 1.34 | 0 | 3 | 1 | 0 |
| TGME49_293340 | ran binding family protein 1, putative | 100 | 24292 | 8.5 | 2 | 3 | 2 | 0 |
| TGME49_311690 | UBA/TS-N domain-containing protein | 100 | 45976 | 9.75 | 0 | 3 | 3 | 0 |
| TGME49_313670 | adaptin N-terminal region domain-containing protein | 89 | 107007 | 4 | 1 | 3 | 5 | 0 |
| TGME49_321650 | hypothetical protein | 100 | 153575 | 3.3 | 1 | 3 | 5 | 0 |
| TGME49_203600 | hypothetical protein | 99 | 47215 | 6.35 | 0 | 2 | 2 | 0 |
| TGME49_216050 | tetratricopeptide repeat-containing protein | 75 | 59519 | 0.765 | 0 | 2 | 1 | 0 |
| TGME49_228760 | hypothetical protein | 87 | 49240 | 4.3 | 0 | 2 | 1 | 0 |
| TGME49_246740 | hypothetical protein | 75 | 64742 | 2.15 | 0 | 2 | 1 | 0 |
| TGME49_247450 | hypothetical protein | 100 | 245873 | 2 | 3 | 2 | 2 | 0 |
| TGME49_288530 | NOL1/NOP2/sun family protein | 100 | 87639 | 3.9 | 0 | 2 | 0 | 0 |
| TGME49_210360 | DEAD (Asp-Glu-Ala-Asp) box polypeptide 41 family protein | 99 | 73229 | 3 | 2 | 1 | 0 | 0 |
| TGME49_219790 | pre-mRNA processing factor PRP3 | 98.5 | 76835 | 2.3 | 0 | 1 | 1 | 0 |
| TGME49_226640 | zinc binding protein, putative | 94 | 14670 | 17.2 | 0 | 1 | 2 | 0 |
| TGME49_240060 | hypothetical protein | 75 | 88534 | 1.35 | 0 | 1 | 1 | 0 |
| TGME49_246340 | DnaJ domain-containing protein | 100 | 95361 | 2.55 | 2 | 1 | 0 | 0 |
| TGME49_289540 | hypothetical protein | 74 | 100518 | 4.7 | 1 | 1 | 7 | 0 |
| TGME49_304680 | ubiquitin family protein | 87.5 | 56734 | 6.3 | 1 | 1 | 0 | 0 |
| TGME49_206670 | hypothetical protein | 61.5 | 218501 | 0.48 | 2 | 0 | 0 | 0 |
| TGME49_293060 | SPRY domain-containing protein | 100 | 80292 | 1.6 | 0 | 0 | 1 | 0 |
| TGME49_306600 | RNA recognition motif-containing protein | 99 | 21205 | 6.9 | 0 | 0 | 1 | 0 |

*Affinity Purified α-SMC1 antibody **Proteins shown on this table were either absent from the control sample and present in the positive samples, or showed 10-fold enrichment in number of unique peptides in at least one of the positive samples as compared to the negative control sample. Note that proteins are ordered according to their abundance of appearance in the affinity Purified α-SMC1 antibody assay. TgSMC1 is marked in green as a reference.

**Table S2: Name and sequences of all primers used in this study**

| Primer Name | Purpose | Sequence (5’🡪3’) |
| --- | --- | --- |
| SMC1_LIC_F (2035) | SMC1 3’ replacement/ endogenous tagging | TACTTCCAATCCAATTTAATGCACCTAGGGACGAAGTCGACGCACCGCTCGACG |
| SMC1_LIC_R (2036) | SMC1 3’ replacement/ endogenous tagging | TCCTCCACTTCCAATTTTAGCCTCCGCATTCTCGGAGGCCAGTAAG |
| LIC_YFP_R (2464) | Screening 3’ replacement with YFP | CGGTGAACAGCTCCTCCGCCCTTGCTCAC |
| LIC_3HA_R (1595) | Screening 3’ replacement with 3HA | GGATAGCCAGCGTAGTCCGGG |
| SMC1_Lic_screening_F (3529) | Screening - SMC1 3’ replacement | TACTTCCAATCCAATTTAATGCAGCTAGCAGCGTTTGCTGCCTCTGCAATCTGTTGAG |
| pAVA_SMC1_C_F (2549) | SMC1 cDNA cloning into pAVA for C-terminal peptide synthesis | GGGTCCTGGTCCGATGTGGGTCTACAGAGAAGAGAAAC |
| pAVA_SMC1_C_R (2548) | SMC1 cDNA cloning into pAVA for C-terminal peptide synthesis | CTTGTTCGTGCTGGGAGGCCAGTAAG |
| Exportin1_LIC_F (2938) | TgExportin1 3’ replacement/ endogenous tagging | TACTTCCAATCCAATTTAATGCAAAGCTTTCAGACCGTCAACCAGAAA |
| Exportin1_LIC_R (2871) | TgExportin1 3’ replacement/ endogenous tagging | TCCTCCACTTCCAATTTTAGCGTCATCGTCTCCTCCACGAACTGTC |
| Exportin1_LIC_ScreenF (3200) | Screening – TgExportin1 3’ replacement | CCAAGGCAGAGCGCGCCTCGGATTT |
| Importin_LIC_F(3284) | TgImportin1 3’ replacement/ endogenous tagging | TACTTCCAATCCAATTTAATGCACGTTGCTGCCACTCGTTAAATCAAC |
| Importin_LIC_R (3285) | TgImportin1 3’ replacement/ endogenous tagging | TCCTCCACTTCCAATTTTAGCGAGACAAACAAAGGGAAGGAGAGGCGT |
| Importin_LIC_Scrn_F (3391) | Screening – TgImportin1 3’ replacement | CGTCTGCACGGAATACTTACGTCTG |
| SUN_LIC_F (3348) | SUN 3’ replacement/ endogenous tagging | TACTTCCAATCCAATTTAATGCACCACTGTACTGTTGATGCGTCTGTG |
| SUN_LIC_R (3349) | SUN 3’ replacement/ endogenous tagging | TCCTCCACTTCCAATTTTAGCCGCACGCTTCTTAGAAATACTTGC |
| SUN_LIC_ScreenF (3393) | Screening – SUN 3’ replacement | GTACGGACAGGCAGAGAGATTCCGT |

- Primer sequences used for amplification of FISH probes can be found in Gissot et. al. 2011[[14](#_ENREF_14)]
